# Supplementary material for: Splice-Junction-Based Mapping of Alternative Isoforms in the Human Proteome
Source: Cell Rep. Author manuscript; Available in PMC 2020 Jan 15. (PMC6961840; doi:10.1016/j.celrep.2019.11.026)
Supplement: 2 [file NIHMS1546469-supplement-2.zip › DF1/PXD006675/SmoothMuscleCells/SmoothMuscleCells_16_GLYR1_ILIDLVWGKLGR.pdf]

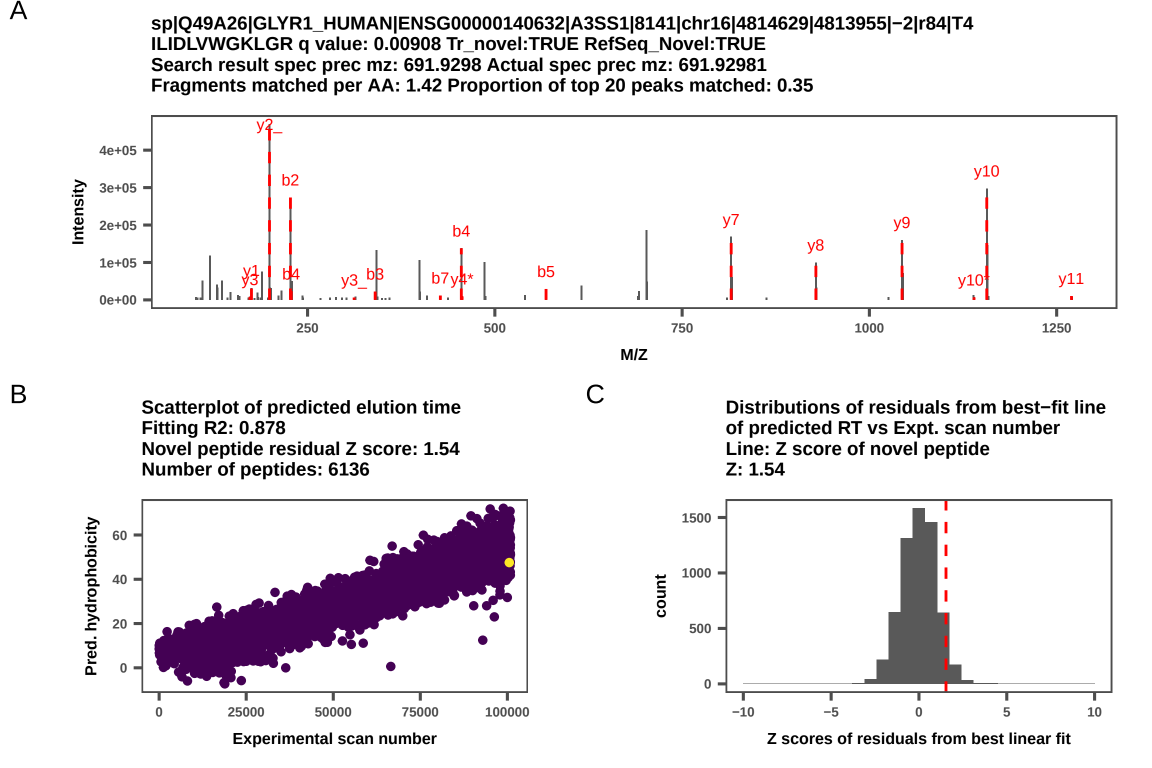
A
sp|Q49A26|GLYR1_HUMAN|ENSG00000140632|A3SS1|8141|chr16|4814629|4813955|−2|r84|T4
ILIDLVWGKLGR q value: 0.00908 Tr_novel:TRUE RefSeq_Novel:TRUE
Search result spec prec mz: 691.9298 Actual spec prec mz: 691.92981
Fragments matched per AA: 1.42 Proportion of top 20 peaks matched: 0.35
Intensity
4e+05
3e+05
2e+05
1e+05
0e+00
y2_
b2
y1
y3
b4
y3_
b3
b7
y4*
b4
b5
y7
y8
y9
y10
y10*
y11
250
500
750
1000
1250
M/Z
B
Scatterplot of predicted elution time
Fitting R2: 0.878
Novel peptide residual Z score: 1.54
Number of peptides: 6136
Pred. hydrophobicity
60
40
20
0
0
25000
50000
75000
100000
Experimental scan number
C
Distributions of residuals from best−fit line
of predicted RT vs Expt. scan number
Line: Z score of novel peptide
Z: 1.54
count
1500
1000
500
0
−10
−5
0
5
10
Z scores of residuals from best linear fit
